# Memory Trace for Fear Extinction: Fragile yet Reinforceable

**DOI:** 10.1007/s12264-023-01129-3

**Published:** 2023-10-09

**Authors:** Ying Liu, Shuai Ye, Xin-Ni Li, Wei-Guang Li

**Affiliations:** Department of Rehabilitation Medicine, State Key Laboratory of Medical Neurobiology and Ministry of Education Frontiers Center for Brain Science, Huashan Hospital, Institute for Translational Brain Research, Fudan University, Shanghai, 200032 China

**Keywords:** Fear extinction memory, Memory trace, Fear relapse, Medial prefrontal cortex, Basolateral amygdala, Ventral hippocampus, Insular cortex, Synaptic adaptations

## Abstract

Fear extinction is a biological process in which learned fear behavior diminishes without anticipated reinforcement, allowing the organism to re-adapt to ever-changing situations. Based on the behavioral hypothesis that extinction is new learning and forms an extinction memory, this new memory is more readily forgettable than the original fear memory. The brain’s cellular and synaptic traces underpinning this inherently fragile yet reinforceable extinction memory remain unclear. Intriguing questions are about the whereabouts of the engram neurons that emerged during extinction learning and how they constitute a dynamically evolving functional construct that works in concert to store and express the extinction memory. In this review, we discuss recent advances in the engram circuits and their neural connectivity plasticity for fear extinction, aiming to establish a conceptual framework for understanding the dynamic competition between fear and extinction memories in adaptive control of conditioned fear responses.

## Introduction

Extinction in neuroscience describes a fundamental physiological phenomenon in which the absence of reinforcement leads to the weakening or disappearance of learned behaviors [1–3]. Extinction serves as an inhibitory learning and memory in which organisms adapt flexibly to constantly changing environments. Extinction has also been identified as a process that underlies the induced behavior changes by psychotherapies for many mental disorders, such as anxiety disorders, post-traumatic stress disorder (PTSD), and drug cravings [4, 5]. Anxiety and other related disorders evoked by trigger cues associated with emotional trauma are typically characterized by the persistence of learned fear. However, fear is not all bad; fear at the physiological level, as a fundamental, cross-species conservative emotion, represents a collection of defensive behavioral responses of an organism when encountering an immediate, imminent, or predictable danger or threat, and is therefore essential for survival in challenging environments [6, 7]. In order to achieve appropriate levels of learned fear, advances in the understanding of fear extinction will hopefully provide benefits for effective treatments of psychiatric disorders characterized by the inability to regulate pathological fear or anxiety [4, 8, 9].

In the laboratory, fear conditioning, which originates from the experience of fear emotion, is the most thoroughly studied memory paradigm [6, 10, 11]. Behaviorally, classical conditioning and operant conditioning have been extensively studied for >100 years. While classical conditioning utilizes instinctive behaviors and can be divided into contextual fear conditioning (CFC) and cued fear conditioning according to different conditioned stimuli (CS) such as context, tone, and aroma, operant conditioning manipulates conscious behavioral tendency by endowing rewards or punishment [7]. Typically, in classical fear conditioning, a CS (like an auditory tone) is paired with an aversive incentive (usually a mild foot electric shock, unconditioned stimulus, US). After fear learning, the presentation of the CS alone generates various conditioned fear responses, such as freezing [12]. However, in the absence of the footshock as a negative reinforcer, repeating exposure to CS alone attenuates the conditioned fear responses, a process termed fear extinction. The efficacy of fear extinction for suppressing learned fear behaviors is encouraging, but it is at least as important to keep in mind the fragile nature of fear extinction. Fear extinction is highly context-dependent, as extinguished fear memory may return in a new, non-extinguished context, a process known as fear renewal [13–16]. Moreover, extinguished fear may re-emerge over time or following an aversive event, processes termed spontaneous recovery and fear reinstatement [16–18], respectively. Thus, extinction is generally thought to result from a new inhibitory learning rather than an erasure of the original fear memory. To fully understand the behavioral features and biological bases of fear extinction, mapping the enduring but dynamic physical changes—memory traces for fear extinction—at multiple levels distributed across whole-brain regions is an inevitable way to go.

Memory trace, also a specific term “engram”, is designated as neural substrates in the brain for storing and recalling memories. In essence, a behavioral experience activates a sparsely distributed population of neurons that undergo persistent changes to become the cellular representations of memory traces, i.e., engram neurons, which are subsequently reactivated by natural cues available at the time of the experience or by artificial manipulation, leading to memory retrieval [19, 20]. Based on these criteria described above for a cell to be the memory engram cell, the memory engram neurons have been defined and identified by using diverse memory paradigms in multiple brain regions [19, 20]. Not only the cellular representations of memory traces but also the dynamic synaptic connectivity between engram neurons as neural correlates dictate the expression of memory. Research on memory traces of fear extinction pale in comparison to studies on the acquisition process of original fear memory, but it indeed began to emerge until very recently. In this review, we discuss manifestations of memory trace for fear extinction, mainly located in a tripartite neural circuit [2] involving the amygdala, prefrontal cortex, and hippocampus, in addition to emerging new circuits engaged in fear extinction [21–26]. In particular, we survey recent advances to address the typically fragile nature of memory traces for fear extinction [16], aiming to conceptualize the trade-off between new learning and original fear memory modification [27]. Finally, we discuss novel reinforceable strategies to enhance the effects of extinction, aiming to understand the success of these approaches with respect to memory traces.

## Fear Extinction as a Dynamic Modification of Fear Memory Trace

### Fear Extinction Reverses Fear-conditioning-activated Excitatory Synaptic Traces

**Fig. 1:**
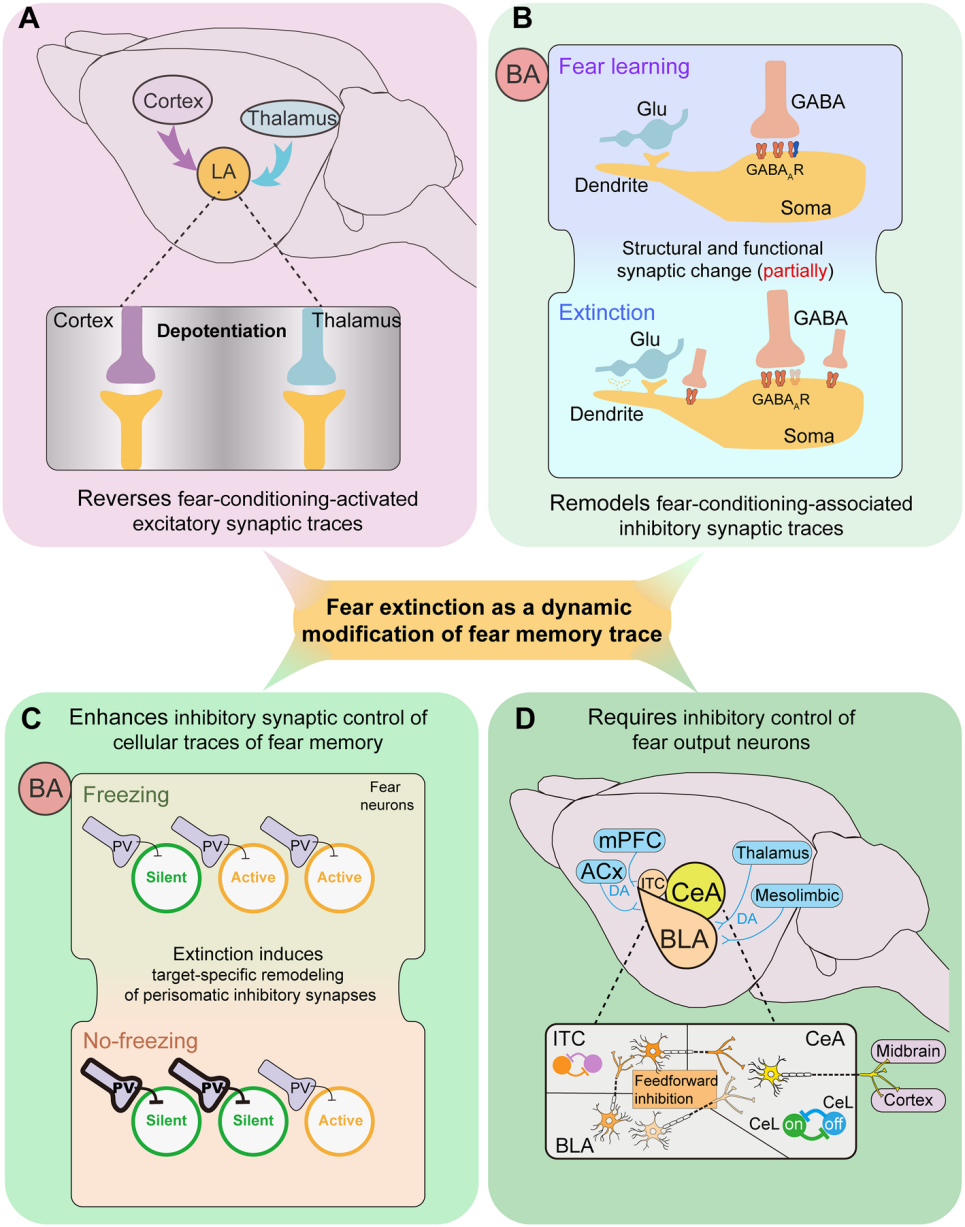
Schematic illustrating fear extinction as a dynamic modification of the fear memory trace. **A** Fear extinction reverses fear-conditioning-activated excitatory synaptic traces. Based on the findings shown in [29, 30], fear extinction involves different forms of depotentiation at LA synapses to tune the synaptic traces of fear memory for the expression of cue-induced non-fear behaviors. **B** Fear extinction remodels fear-conditioning-associated inhibitory synaptic traces. Based on the finding shown in [39], fear conditioning and extinction sculpt inhibitory synapses to regulate the inhibition of active neuronal networks to tune the amygdala circuit responses to threats. **C** Fear extinction enhances inhibitory synaptic control of cellular traces of fear memory. Based on the finding shown in [40], fear extinction entails target-specific alterations in perisomatic inhibitory synapses to sculpt activation patterns in fear circuits through which conditioned fear responses can be reduced. **D** Fear extinction requires inhibitory control of fear output neurons. ACx, auditory cortex; BA, basal amygdala; BLA, basolateral amygdala; CeA, central amygdala; CeL, lateral division of central amygdala; DA, dopamine; GABA, γ-aminobutyric acid; GABA_A_R, type A GABA receptor; Glu, glutamate; LA, lateral amygdala; ITC, intercalated; mPFC, medial prefrontal cortex. Please see the text for more details

Fear memory-based adaptive behaviors depend on the modification of synaptic strengths onto lateral amygdala (LA) neurons through cellular mechanisms such as long-term potentiation (LTP). Here synaptic plasticity allows subsets of neurons to be recruited as engram neurons during fear learning and is critical for memory retrieval [28]. As a behavioral counteraction to fear memory retrieval, fear extinction is considered a macroscopic manifestation of synaptic plasticity in contrast to LTP, i.e., long-term depression (LTD). More specifically, depotentiation, the reversal of fear conditioning-induced synaptic potentiation, has been proposed as a cellular mechanism for fear extinction [29, 30]. As supportive *ex vivo* evidence, excitatory synapses to LA from thalamic [29] and cortical [30] pathways both underwent a net depression of fear conditioning-induced potentiation. Moreover, extinction training returned the enhanced efficacy of synapse onto LA in fear-conditioned rats to baseline and occluded further induction of depotentiation. Mechanistically, depotentiation of the thalamic pathway is expressed in a postsynaptic manner, requiring group I metabotropic glutamate receptor (mGluR) activity and AMPA receptor (AMPAR) internalization [29], whereas depotentiation of the cortical pathway is expressed in a presynaptic manner, requiring NMDA receptor (NMDAR) and group II mGluR activity [30]. These results suggest that fear extinction involves different forms of depotentiation at LA synapses (Fig. 1A) to tune the synaptic traces of fear memory for the expression of cue-induced non-fear behaviors.

To demonstrate the causal link between synaptic potentiation or depression and memory expression, Nabavi *et al.* took advantage of optogenetic engineering approaches to show that fear memory can be inactivated and reactivated by LTD and LTP of auditory inputs targeting LA [31]. Conditioning animals to associate foot shocks with optogenetic stimulation of auditory inputs originating from both the auditory thalamus and auditory cortex resulted in an LTP of optically driven synaptic response in LA and optogenetically driven conditioned responses indicative of memory for aversive stimuli. Subsequent optogenetic delivery of LTD protocol to the auditory input inactivated memory of the shock, whereas subsequent optogenetic delivery of LTP protocol to the auditory input reactivated memory of the shock. Although conditioned responses can be extinguished by repeated exposure to optical CS, the failure of the optical LTP protocol to restore conditioned responses argues against the notion that extinction is a merely weakening of synapses potentiated during paired conditioning. These results support a causal relationship between synaptic traces and conditioned fear behaviors, but fear extinction cannot simply be attributed to the reversal of synaptic traces activated by fear conditioning.

Using a discriminative fear learning paradigm and a behavioral activity-dependent neuronal labeling approach, it was further shown that LTP is expressed selectively in the CS-specific auditory pathways to the LA [32, 33]. Consistent with the observation in the non-discriminative fear conditioning protocol discussed before [31], optogenetically induced depotentiation of the CS-specific auditory pathways to the LA suppressed conditioned fear responses to the CS in the discriminative fear learning paradigm [32, 33]. By contrast, synapses in the CS-specific auditory pathways remained potentiated after fear extinction [32], again suggesting that extinction is a distinct process from the depotentiation-induced reduction of conditioned fear responses. Thus, input-specific synaptic potentiation or depotentiation of LA is sufficient to determine the expression of fear memory or not in a sensory cue-specific manner; however, reversal of synaptic traces of fear memory in the LA is not necessary for fear extinction to allow similar control of adaptive fear responses.

**Fig. 2:**
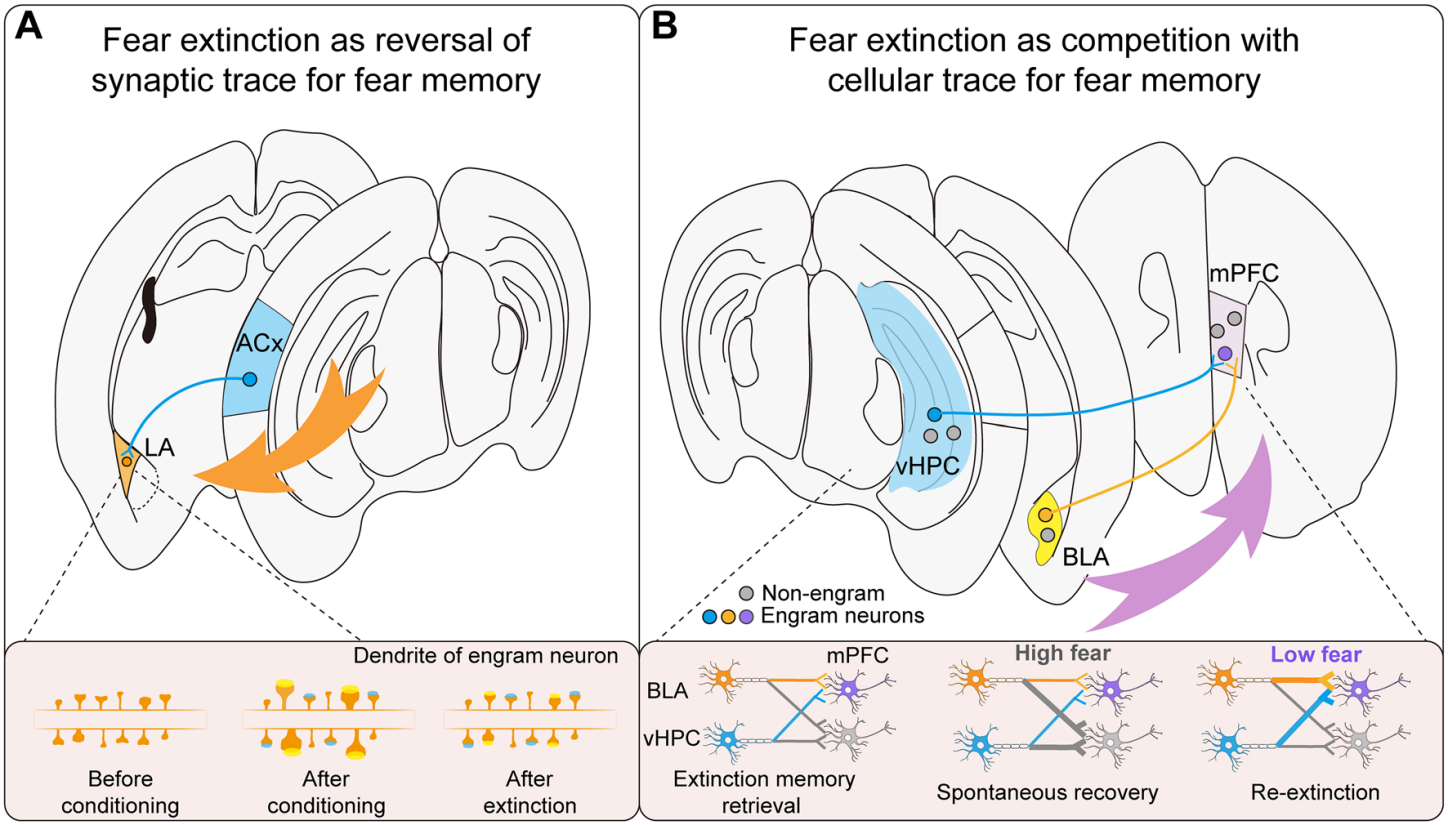
Schematic representation of fear extinction as the reversal of the synaptic trace for fear memory or competition with cellular trace for fear memory. **A** Fear extinction reverses the synaptic trace for fear memory. Based on the finding shown in [27], the synaptic trace, represented by activated spines, is enhanced by fear conditioning and subsequently decreased by extinction. **B** Schematic representation of the independent extinction engram construct, which consists of multiple synaptic traces generated by extinction learning and designed to compete with fear memory traces. Based on the finding shown in [16], the unidirectional connectivity of new memory engram neurons from BLA and vHPC to mPFC is established during the formation of extinction memory, providing a tripartite construct of circuitry for the extinction memory encoding and storage. Dynamic remodeling of specific engram connectivity dictates both the validity and instability of fear extinction memory, allowing for a longitudinal transformation of memory fate from fear to extinction, relapse, and re-extinction. ACx, auditory cortex; BLA, basolateral amygdala; mPFC, medial prefrontal cortex

However, utilizing an input-specific and activity-dependent spine labeling technique called dual-eGRASP (enhanced green fluorescent protein reconstitution across synaptic partners) [34], researchers have reported that specific synapses originating from auditory fear conditioning-activated neuronal ensembles in the auditory cortex (ACx) exhibited enhanced and reduced spine morphology after fear conditioning and fear extinction, respectively (Fig. 2A) [27]. Notably, when re-conditioning with the same tone and shock was performed, the fear-extinction-induced reduction in synaptic ensembles was restored, indicating a correlation between the spine morphology of activated synaptic ensembles and the fear memory state [27]. Moreover, employing the dual-eGRASP strategy in combination with *in vivo* two-photon microscopy, the same group of researchers observed that while synaptic connections between engram populations were enhanced alongside synaptogenesis within the hippocampal network, extinction learning specifically correlated with the disappearance of synapses from hippocampal CA3 to CA1 engrams [35].

Another study using a contextual fear conditioning paradigm demonstrated that the consolidation of remote memories in mice was associated with the progressive strengthening of excitatory connections between the medial prefrontal cortex (mPFC) engram neurons activated during learning and reactivated during remote memory retrieval. In contrast, the extinction of remote memories weakened these synapses [36]. These findings suggest that fear extinction, although not always consistent, does result in the reversal of fear-conditioning-activated excitatory synaptic traces, emphasizing the importance of precise mapping techniques to accurately identify the relevant subset of synaptic structure and function. It is worth noting that fear extinction involves two opposing and complementary mechanisms known as “unlearning” and “new learning” [3, 37, 38]. Therefore, it is likely that some synapses undergo de-potentiated during fear extinction, as discussed above, while others may be involved in new forms of learning that suppress the originally formed fear memory, as discussed below.

### Fear Extinction Remodels Fear-Conditioning-Associated Inhibitory Synaptic Traces

In addition to excitatory glutamatergic synapses in the amygdala, which represent different fear memory states, fear conditioning also entails long-lasting plasticity of GABAergic synapses onto pyramidal neurons [39]. Auditory fear conditioning induces structural and functional plasticity of GABAergic synapses in the basal amygdala (BA), which is associated with an increase in the fraction of synaptic GABA_A_ receptors containing the α2 subunit [39]. These learning-induced inhibitory synaptic changes were also partially reversed by fear extinction (Fig. 1B) [39], which implies that fear conditioning and extinction sculpt inhibitory synapses to regulate the inhibition of active neuronal networks to tune the amygdala circuit responses to threats.

### Fear Extinction Enhances Inhibitory Synaptic Control of Cellular Traces of Fear Memory

As an alternative plasticity to counteract learning-induced changes in neuronal activity and connectivity within fear circuits, fear extinction typically enhances inhibitory synaptic control of the cellular traces of fear memory. Using a Fos-based transgenic mouse, contextual fear extinction was found to specifically silence a subset of BA excitatory neurons that were previously activated during fear conditioning (i.e., cellular traces of fear memory) [40]. Silencing of cellular traces of fear memory is achieved by extinction-induced target-specific remodeling of perisomatic inhibitory synapses originating from parvalbumin (PV)- and cholecystokinin-positive interneurons [40]. Thus, fear extinction entails target-specific alterations in perisomatic inhibitory synapses to sculpt activation patterns in fear circuits through which conditioned fear responses can be reduced. Interestingly, homeostatic adaptations of cellular traces for contextual fear memory in hippocampal granule cells can be triggered by sustained neural activity, as evidenced by a decrease in excitatory synapses and an increase in inhibitory synapses, which can facilitate fear extinction [41]. Together, these results suggest that fear extinction alters the balance between inhibitory and excitatory synaptic inputs to the cellular traces of fear memory (Fig. 1C) to allow for adaptive control of conditioned fear behaviors.

### Fear Extinction Depends on Inhibitory Control of Fear Output Neurons

Fear extinction is associated with altered levels of synaptic inhibition of fear output neurons in the central amygdala (CeA). CeA contains lateral (CeL) and medial (CeM) subregions, in addition to multiple functionally and genetically defined cell types that interact to calibrate the level of fear response, thus CeA inhibitory microcircuits serve as a crucial element in mediating fear extinction [23, 42]. Besides, the increased inhibition of CeA fear output neurons results from the potentiation of fear input synapses to intercalated (ITC) amygdala neurons that project to the CeA [43]. Paracapsular ITC cells act as a subset of GABAergic interneurons forming a network around the basolateral amygdala (BLA) and provide feedforward inhibition to BLA and CeA [44], and are therefore necessary for fear extinction [45]. ITC neurons exert control over the acquired fear behaviors relying on their cognitive, sensory, and emotional inputs from the prefrontal cortex, auditory cortex and thalamus, and mesolimbic dopaminergic afferents, respectively [46–48]. Distinct inhibitory clusters of ITCs play contrasting roles in the acquisition and retrieval of fear extinction [49]. These ITC clusters antagonize one another through mutual synaptic inhibition and differentially access functionally distinct cortical- and midbrain-projecting amygdala output pathways [49]. Overall, inhibitory control in the amygdala represents a substrate during fear extinction (Fig. 1D) for achieving an appropriate balance between avoiding threatening predictive stimuli and suppressing excessive defensive behavior following uneventful encounters.

The mPFC exerts a top-down control of fear and extinction behaviors [21, 50]. Of these, the infralimbic subdivision of mPFC (IL/mPFC) sends a monosynaptic glutamatergic pathway to BLA, which in turn drives the activity of ITC amygdala neurons to inhibit the CeA fear output neurons [43, 48]. As a result, selective stimulation of the IL-BLA pathway facilitated fear extinction but not retrieval [51]. Conversely, silencing the IL-BLA pathway impaired fear extinction and reduced extinction-associated amygdala activity [51]. Thus, mPFC inputs to BLA instruct fear extinction. Outside the classical IL-amygdala circuit, IL projections to the paraventricular thalamus (PVT) also mediate retrieval of fear extinction [52]. The thalamic nucleus reuniens (RE) is another crucial structure mediating prefrontal top-down inhibitory control for fear extinction [21, 53, 54]. Extinction training or retrieval testing increases the activity of RE neurons, while inactivation of either RE or its inputs from the mPFC impairs encoding and retrieval of fear extinction [21]. In addition, different IL projections exert opposing effects in modulating fear extinction, with IL to the lateral septum (LS) and IL-CeA projections suppressing and promoting fear extinction, respectively [55]. Together, the mPFC inputs to different target regions regulate specific aspects of fear and extinction through top-down control mechanisms.

To unravel the synaptic encoding mechanisms of fear extinction retrieval, Cho *et al*. showed that fear extinction reduced the efficacy of excitatory glutamatergic synaptic transmission in projections from mPFC to BLA without altering that of the feedforward inhibitory responses, thereby shifting the excitation/inhibition balance in these projections towards more inhibition. Moreover, priming stimulation of mPFC projections induced heterosynaptic inhibition in auditory cortical inputs to the BLA. These synaptic mechanisms diminish the ability of projections from the mPFC to drive BLA activity while retaining the ability of ITC neurons to inhibit the CeA fear output neurons [56], which could contribute to the encoding of fear extinction retrieval.

## Memory Traces Emerging from Extinction Learning

### Extinction Activates a Distinct Set of Neurons from the Fear Memory Cellular Traces

In addition to being a dynamic modification of fear memory traces, fear extinction is also thought to be a switch off of conditioned fear by distinct neuronal circuits. Herry *et al*. used the opposite behavioral states of fear extinction and its context-dependent renewal to electrophysiologically identify “fear neurons” and “extinction neurons” in BA that exhibit selective increases in CS^+^-evoked spike firing associated with fear conditioning and extinction training, respectively [57]. Bidirectional transitions between high and low fear states are triggered by a rapid switch in the balance of activity between these two distinct neuronal populations [57]. According to the long-range connectivity, Senn *et al*. further defined subpopulations of BA neurons as fear neurons and extinction neurons, respectively [58]. The BA projection neurons targeting the prelimbic subdivision of mPFC (PL/mPFC) are active during the states of high fear, whereas BA neurons targeting the IL/mPFC are recruited and exhibit cell-type-specific plasticity during fear extinction [58], suggesting one group of BLA neurons signals fear conditioning and expression (“fear neurons”) and the other signals extinction (“extinction neurons”). Moreover, a set of molecular-defined neurons expressing neurotensin receptor 2 (NTSR2) within BA has been identified as a putative population of “extinction neurons” [59]. These results suggest that extinction activates a distinct set of neurons from the fear memory cellular traces to specifically execute the “fear-off” behaviors.

Historically, Richard Semon proposed that an experience activates a subset of cells that undergo offline, persistent chemical and/or physical changes to become an “engram”. Through activity-dependent genetic tagging, such as the approach termed targeted recombination in active populations (TRAP) [60, 61], to gain genetic access to neurons activated by defined stimuli, researchers have begun to define the engram as the basic unit of memory [20, 62]. To investigate the cellular representations of fear extinction memory, Lacagnina *et al*. utilized activity-dependent neuronal tagging to demonstrate that extinction training suppresses the reactivation of contextual fear engram neurons while activating a second engram cell ensemble, a putative extinction engram in the hippocampal dentate gyrus (DG) [63]. Optogenetic inhibition of neurons active during extinction training (i.e., extinction engram neurons) increased fear after extinction training, whereas stimulation of extinction engram neurons suppressed fear and prevented spontaneous recovery. In stark contrast, silencing neurons active during fear conditioning (i.e., fear engram neurons) reduced spontaneous recovery of fear, whereas optogenetic stimulation of fear engram neurons increased conditioned fear behaviors. In addition, contextual fear extinction training also elicits functional engram cell ensembles in the retrosplenial cortex (RSC), the generation of which requires adult hippocampal neurogenesis [64]. These results indicate that the hippocampus and related brain structures generate a fear extinction engram and that interactions between fear and extinction engrams govern suppression and relapse of fear after extinction [63].

To further map the location and nature of the newly formed extinction memory, Zhang *et al*. showed that a contextual fear extinction memory engram is formed and stored in a genetically distinct BLA neuronal population [65] thought to be associated with positive valence [66, 67]. Activation of fear extinction engram neurons and natural reward-responsive neurons overlap significantly in the BLA [65]. Furthermore, these two neuronal subsets are mutually interchangeable in driving reward behaviors and fear extinction behaviors [65]. Thus, fear extinction memory is a newly formed reward memory [68] by recruiting amygdala reward neurons as engram neurons for fear extinction memory.

### Reciprocal Inhibition Between Fear and Extinction Memory Cellular Traces

**Fig. 3:**
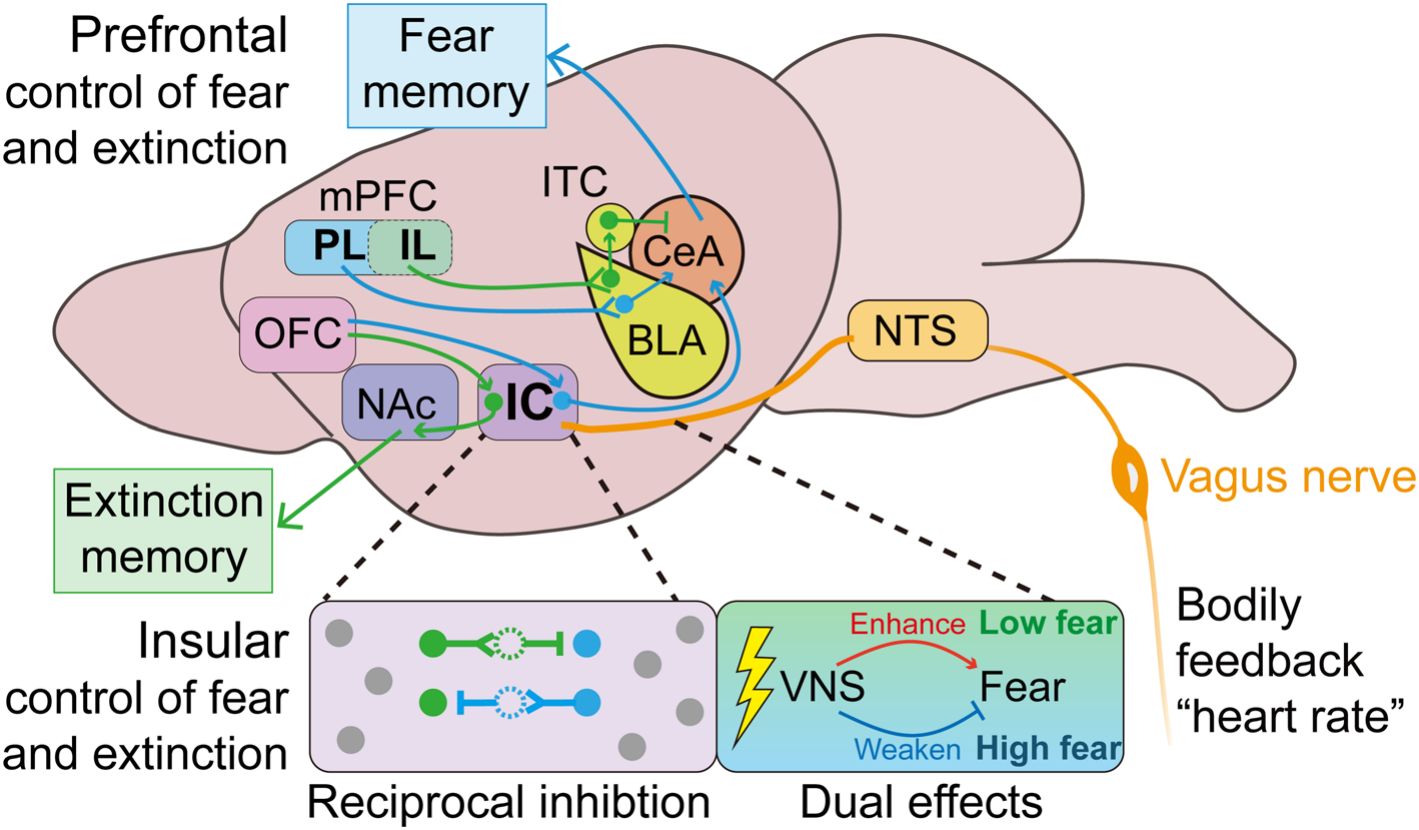
Comparison between prefrontal and insular control of fear and extinction memories. *Upper* Connections between the mPFC and amygdala encode fear and extinction memories. The PL/mPFC is thought to mediate fear responses (blue), whereas the IL/mPFC mediates extinction (green). For the expression of fear memory, PL inputs to the BLA drive glutamatergic neurons that project to the CeA, and outputs from the CeA drive the fear response. For the expression of extinction memory, IL inputs to the BLA drive glutamatergic neurons that project to the ITC, which mediates feedforward inhibition of neurons in the CeA. *Lower* IC circuits as an executive gateway to decipher fear or extinction memory *via* distinct subcortical pathways. Based on the finding shown in [22], there are two distinct populations of IC neurons, defined by their differential long-range connectivities, coordinate respective fear and extinction memories. IC-CeA and IC-NAc projectors encode fear and extinction memories, respectively. The reciprocal inhibitions of IC-CeA and IC-NAc projectors *via* local interneurons drive memory-guided behaviors in opposite directions, and their activities undertake distinct modifications during threat and extinction learning. Moreover, the orbitofrontal cortex (OFC)→IC→NAc circuit selectively engages extinction memory and thereby strengthens the specificity of distinct populations of IC neurons defined by their long-range connectivity. Based on the findings shown in [136, 138], the IC integrates predictive sensory and interoceptive signals to provide graded and bidirectional teaching signals that gate fear extinction and tune emotional or affective states. BLA, basolateral amygdala; CeA, central amygdala; IC, insular cortex; IL, infralimbic subdivision; ITC, intercalated; mPFC, medial prefrontal cortex; NAc, nucleus accumbens; NTS, nucleus tractus solitarius; OFC, orbitofrontal cortex; PL, prelimbic subdivision

Conceptually, fear and extinction memories are acquired, stored, and modulated by engram cell ensembles distributed throughout the brain. A fundamental question in fear and extinction memory processing is whether and how these two memories, which lead to opposite behaviors and are thought to be encoded by different engram cell ensembles as cellular traces, compete. Beyond the classical regions involved in fear and extinction memories, such as the mPFC, BLA, and ventral hippocampus (vHPC), we recently found that fear and extinction memories are processed by two distinct subpopulations of projection neurons in close proximity within the insular cortex (IC), targeting the CeA and nucleus accumbens (NAc), termed as IC-CeA and IC-NAc projectors, respectively [22]. Of note, IC-CeA and IC-NAc projectors mutually inhibit each other by recruiting local intracortical interneurons [22]. Fear- and extinction-learning oppositely modulate such reciprocal inhibition: fear conditioning enhances inhibitory inputs from fear cell engram ensembles (i.e., IC-CeA projectors) to extinction cell engram ensembles (i.e., IC-NAc projectors), whereas extinction enhances inhibitory inputs of opposite routes [22]. The mutually inhibitory motif has also been identified between different ITC amygdala clusters [49], in addition to fear and extinction engram cell ensembles in other brain regions [65], to orchestrate a distributed neural circuitry that regulates the switch between high- and low-fear states in turn. Such a circuit motif could amplify small differences in input to an all-or-none output pattern, providing a “winner-take-all” mechanism that could increase signal-to-noise to generate robust circuit outputs and associated behavioral states [69, 70]. Overall, competition between fear and extinction memories can be achieved by mutual inhibitory circuitry between their corresponding engram cell ensembles (Fig. 3) to ensure proper expression of the dominant memory trace and inhibition of the other.

### Extinction Establishes Ascending Plasticity for Top-Down Control of Fear Memory

According to the “new learning” hypothesis, fear extinction requires multiplex plasticity of synaptic connections in the tripartite neuronal circuit consisting of mPFC, BLA, and vHPC. As discussed above, the mPFC exerts top-down control over fear and extinction memories [21, 50]. The IL/mPFC plays a central role in the acquisition of fear extinction memories [71], where the adaptive changes associated with extinction learning, such as NMDAR-dependent bursting [72], are required for the consolidation of fear extinction memory. In contrast, the adjacent PL/mPFC is essential for sustained fear expression and resistance to extinction [73, 74], although a direct synaptic connection from PL/mPFC to IL/mPFC has also been identified to promote fear extinction [75]. The mPFC is extensively modulated by the ascending bottom-up systems associated with fear extinction. As one of the major inputs to the mPFC, the vHPC promotes fear extinction through brain-derived neurotrophic factor (BDNF), which is thought to be released from vHPC projections and acts at IL/mPFC neurons [76, 77]. Specifically, fear extinction learning leads to increased expression of BDNF in the hippocampus, with an emphasis on its ventral part. Furthermore, BDNF infusion in the hippocampus sufficiently induces the extinction of conditioned fear, which is prevented by the coadministration of BDNF-inactivating antibodies in the IL/mPFC [76]. Thus, hippocampal-prefrontal BDNF signaling is a key molecular substrate for fear extinction.

To further unravel the underlying molecular substrates that coordinate the dynamics of synaptic connections associated with fear extinction, we recently identified an involvement of acid-sensing ion channel 1a (ASIC1a) specifically in vHPC, but not BLA or mPFC, in regulating fear extinction behavior and extinction learning-induced hippocampal-prefrontal synaptic plasticity [78]. ASIC1a serves as the major H^+^ receptor in the central nervous system, sensing extracellular pH fluctuations and mediating cation influx. In addition, ASIC1a has been proposed to be a protonergic synaptic receptor that senses protons released from presynaptic vesicles during synaptic transmission, thereby contributing to synaptic plasticity [79–81]. Under the scenario with fear extinction, ASIC1a in vHPC neurons drives BDNF expression and mediates antegrade BDNF signaling at the vHPC→IL/mPFC projections, which enhances the postsynaptic NMDAR function. Electrophysiologically, a number of extinction-related plasticity features in hippocampal-prefrontal correlates, including changes in transmitter release probability and postsynaptic NMDAR activity [82], are opposite in vHPC→IL/mPFC *versus* vHPC→PL/mPFC synapses, but are consistently abolished in ASIC1a-deficient animals [78]. Therefore, the ASIC1a-BDNF signaling cascade in vHPC contributes to fear extinction in a manner dependent on the fine-tuning of hippocampal-prefrontal connections.

The mPFC and amygdala form a critical bidirectional highway for processing complex emotional information. As discussed above, the mPFC recruits not only excitation but also inhibition in the amygdala to encode fear extinction in a top-down manner [56]. The mPFC, in turn, also integrates the message from the amygdala to signal emotional valence across a range of behaviors and motivational drives in a bottom-up manner [83–85]. Klavir *et al*. showed that amygdala inputs to both PL/mPFC and IL/mPFC convey the association between a stimulus and aversion [84]. They developed an optogenetic protocol with high-frequency stimulation (HFS) to reversibly depress BLA input to the mPFC in mice, allowing temporally specific down-regulation in the BLA drive of mPFC. Suppression of BLA input to either the PL/mPFC or IL/mPFC, prior to either fear learning or fear extinction, reduces fear associations by attenuating consolidation of subsequent defensive behavior to the aversive stimulus or by promoting the extinction of defensive behavior, receptively [84]. Thus, amygdalar inputs to both the PL/mPFC and IL/mPFC convey aversive information that is then likely further processed in the mPFC to either enhance [86, 87] or extinguish [56] fear associations back in the amygdala.

### Multiple Synaptic Traces Form an Engram Construct for Extinction

Similar to fear memory [20, 88], fear extinction memory is believed to rely on an engram complex, which comprises functionally connected engram cell ensembles distributed across multiple brain regions, with each ensemble representing a specific aspect of the memory. In the auditory extinction paradigm, our research employed an activity-dependent neuronal labeling strategy to uncover the presence of extinction engram neurons in different anatomical locations [16]. Notably, these engram neurons form a tripartite neuronal circuit involving the mPFC, BLA, and vHPC. Within this circuit, there are directional synaptic engram connections from the BLA and vHPC to the mPFC that contribute to the establishment of interregional engram circuits specifically involved in extinction memory (Fig. 2B). Furthermore, it is noteworthy that the mPFC extinction engram neurons also receive synaptic inputs from the mediodorsal nucleus of the thalamus (MD) and the ventral tegmental area (VTA). The MD is considered to play a regulatory role in fear extinction [89], while the VTA is implicated in the generation of bottom-up prediction error signals [90, 91]. These inputs suggest that the formation of extinction memory engrams may involve the integration of multiple bottom-up signals by the mPFC engram neurons. Thus, it is plausible that mPFC engram neurons receive different synaptic traces to consolidate various bottom-up signals and construct the complex engram network underlying fear extinction memory.

Based on the above findings, we propose a working model that illustrates the directional engram connectivity within the engram complex involved in extinction memory. In this model, mPFC engram neurons are postulated to act as a crucial convergence locus for at least two types of information: safety context information carried by vHPC engram neurons and positive emotional valence information carried by BLA engram neurons [16]. Subsequently, this information is likely subjected to further processing through a top-down inhibitory signal from “extinction” engram neurons in the mPFC, which targets “fear” neurons in the amygdala [50, 56]. Additionally, another top-down signal from the mPFC to the RE may mediate the suppression of conditioned fear responses towards an extinguished CS within the extinction context [21].

## Forgetting of Extinction Memory: Vulnerable Engram Construct

### Inaccessible State of Extinction Engram Construct Underlies Return of Extinguished Fear

Compared to the original fear memory, the fear extinction memory is more prone to forgetting and rapidly loses its control over behavior, rendering it inaccessible. Typically, extinguished fear tends to return [2, 17] through phenomena known as fear renewal, spontaneous recovery, or fear reinstatement, which occur in a contextual, temporal, or aversive-dependent manner, respectively. However, the cellular mechanisms underlying the inherent forgetting [92] of extinction memory remain unclear. In conjunction with the dynamic and unstable nature of fear extinction memory, the engram construct involved in extinction, specifically the directional synaptic engram connectivity from the BLA to mPFC or vHPC to mPFC [16], undergoes dynamic changes in synaptic strength.

Unlike the temporal evolution of memory ensembles and circuit reorganization that facilitate the retrieval of recent to remote fear memories [61, 93, 94], the mPFC extinction memory engram neurons progressively become silent over time, contributing to the spontaneous recovery of extinguished fear [16]. However, additional extinction training facilitates the retrieval of extinction memory through natural cues and results in an electrophysiological enhancement of presynaptic transmitter release probability and postsynaptic AMPAR/NMDAR ratio specifically in mPFC engram neurons, compared to non-engram neurons [16]. This indicates that there is selective synaptic potentiation from BLA or vHPC engram neurons to mPFC engram neurons, enabling the establishment of the fear-extinguished state. Notably, optogenetic induction of LTP in the BLA→mPFC and vHPC→mPFC engram connectivity restores the retrieval of extinction memory when presented with natural cues that would otherwise trigger the spontaneous recovery of extinguished fear [16]. These findings align with recent research suggesting that NMDAR-dependent synaptic potentiation in mPFC engram neurons [95] represents mnemonic information associated with extinction.

In summary, spontaneous recovery shifts the extinction engram construct from an accessible state to an inaccessible state, while additional extinction training or optogenetic induction of LTP restores the directional engram connectivity and prevents the return of fear. Thus, the dynamic remodeling of the engram construct underlies the forgetting of extinction memory.

### Re-emerging Fear Memory Engrams Underlie Fear Relapse After Extinction

Based on the competition between fear and extinction memories discussed above, it remains an open question whether and how fear memory engram neurons contribute to fear relapse. More specifically, analogous to fear conditioning, it is also unclear how synaptic plasticity is implemented into fear relapse after extinction. For auditory fear conditioning, the LA is a locus of convergence for auditory (i.e., CS) and somatosensory (i.e., US) information and is a plausible site for CS-US association by recruiting distinct synaptic projections [6]. Consistent with this idea, we identified the synaptic mechanisms underlying context-dependent relapse of extinguished auditory fear memory (i.e., fear renewal), in which fear renewal exploits the associativity rule of Hebbian learning and memory [96] by linking presynaptic plasticity of two independent inputs in the LA [15]. This presynaptic associativity of convergent inputs from the ACx and vHPC, namely the coincident detection of auditory tone-related ACx→LA and context-dependent vHPC→LA pathways, respectively, underlies the reactivation of LA engram neurons active in fear learning to allow fear renewal [15]. Thus, fear renewal represents a particular “learning” process relying on the synaptic associativity of auditory and contextual pathways into LA.

Regarding the involvement of the vHPC in fear renewal and its broader neural projections, studies have indicated that the vHPC sends monosynaptic projections to the CeA [14] and establishes a strong feedforward inhibitory circuit to the IL/mPFC [97], both of which are necessary for fear renewal. Furthermore, animals that undergo fear renewal exhibit preferential activation of vHPC neurons that project simultaneously to both the BA and the PL/mPFC, compared to those subjected to an extinction test [98]. In the vHPC→LA pathway, although it plays a selective role in fear renewal, it does not encode the cued fear memory in a particular context [15]. This suggests that fear renewal is not simply a reactivation of the same pathway associated with the initial fear response.

In a study investigating fear reinstatement, a type of fear relapse in which the extinguished fear response to the CS returns after re-exposure to the US alone, Zaki *et al* provided evidence suggesting that fear relapse triggers a partial reactivation of the original fear memory engram [99]. Specifically, they observed that the engram neurons active during contextual fear conditioning in the hippocampal DG exhibited decreased activity during extinction but were reactivated following the reinstatement of contextual fear [99]. This finding in fear reinstatement aligns with the research on spontaneous recovery, another form of relapse, which demonstrated that extinction suppresses fear-related activity in the DG, but these activity patterns are reinstated during spontaneous recovery [63]. Behaviorally, optogenetic inactivation of the neuronal ensembles active during fear conditioning in the DG was sufficient to disrupt fear expression during both fear reinstatement [99] and spontaneous recovery [63]. These findings collectively suggest that fear relapse relies on the partial reactivation of the cellular engram associated with the original fear memory, although the specific synaptic mechanisms underlying engram reactivation still need to be further elucidated.

### Experience-dependent Interregional Resonance Dictates the Retrieval of Fear and Extinction Memories Following Fear Extinction

Circuit oscillations, which arise from and regulate cellular and synaptic behaviors, enable rapid and flexible transitions between large-scale network states [100]. Fear memory and extinction memory, representing learned threat and safety, respectively, are associated with distinct oscillatory states in the BLA and mPFC [28]. Through fear extinction learning, the network of PV-expressing interneurons undergoes remodeling, allowing for competition between the extinction memory circuit and the fear memory circuit [40, 101]. This competition is reflected in the contrasting behaviors of fear and extinction memory engram neurons within the BLA and the negative correlation between the oscillations in the 3–6 Hz and 6–12 Hz frequency ranges [101]. Following extinction, this competition leads to the suppression of fear engram neurons and a fear-associated 3–6 Hz oscillation in the BLA [101]. In the absence of such competition, fear engram neurons become activated, fear-associated 3–6 Hz oscillations in the BLA increase, and the coherence between the BLA and mPFC shifts toward the 3–6 Hz range, indicating the recurrence of fear expression [101]. Overall, the 3–6 Hz oscillatory activity in the BLA and the engagement of the BLA→mPFC circuit serve as interregional mechanisms that inform the return of fear following extinction learning.

To investigate how interregional oscillatory activity influences the retrieval of competing fear and extinction memories, Ozawa *et al*. employed optogenetic techniques to induce endogenous oscillatory activity by stimulating PV interneurons in the BLA during the retrieval of contextual fear and extinction memories [102]. The exogenously induced 4 Hz oscillations (falling within the 3–6 Hz range) and 8 Hz oscillations (falling within the 6–12 Hz range) in the BLA enhance and suppress conditioned freezing behavior, respectively, in an experience- and context-specific manner [102]. Furthermore, these oscillations recruit distinct functional neuronal ensembles within the BLA [102]. Simultaneous manipulation of the BLA and mPFC with experience-dependent 4 Hz resonance across the BLA-mPFC circuit at the network level supports fear memory retrieval after extinction training [102]. Thus, local and interregional experience-dependent resonance plays a critical role in facilitating the retrieval of fear memory following extinction.

## Reinforcing Extinction Memory: Deconstruction of Fear Memory Trace *vs* Reconstruction of Extinction Memory Trace

### Boundary Conditions for Fear Extinction: Implications for PTSD Pathophysiology

Anxiety disorders and PTSD are characterized by the persistence of learned fear and are closely associated with deficits in fear extinction [8]. It is worth noting that the susceptibility of normal fear conditioning to a rapid extinction process in laboratory rodents contradicts the duration of PTSD in its delayed and chronic forms, calling for the existence of boundary conditions of resistance to extinction in PTSD patients [103]. These boundary conditions refer to the circumstances under which extinction fails to attenuate fear memory. Further exploration and research into these boundary conditions will provide valuable insights into the mechanisms of pathological extinction and the development of therapeutics for individuals with PTSD.

Pitman proposed the concept of superconditioning, suggesting that the strength of the initially conditioned memory serves as a boundary condition for fear extinction. According to this theory, stress hormones released in response to particularly traumatic events enhance memory consolidation, leading to overconsolidation and resistance to extinction [104]. Additionally, animal models of PTSD have utilized fear conditioning following stress induction to mimic the disorder [105]. For example, subjecting animals to a single prolonged stress prior to contextual fear conditioning has been shown to impair fear extinction [106], resembling the characteristics of PTSD. In a study exploring the biological connection between stress and PTSD, highly traumatized women exhibited elevated blood levels of pituitary adenylate cyclase-activating polypeptide (PACAP) and single-nucleotide polymorphisms in its receptor (PAC1R) gene, which were associated with a diagnosis of PTSD and with the extent of fear conditioning responses [107]. Female mice subjected to acute stress consistently displayed impairments in fear extinction due to dysregulation of PACAP-PAC1R signaling in the ventromedial hypothalamus [108]. In summary, the chronicity and severity of PTSD symptoms compared to normal fear conditioning can be attributed to the interplay between extinction deficits, likely influenced by stress as a major factor and enduring memory traces of the trauma event. Therefore, it is crucial to conduct in-depth neurobiological investigations [9, 109] to unravel the boundary conditions of fear extinction.

### Retrieval-extinction Procedure to Destruct the Original Fear Memory Trace

**Fig. 4:**
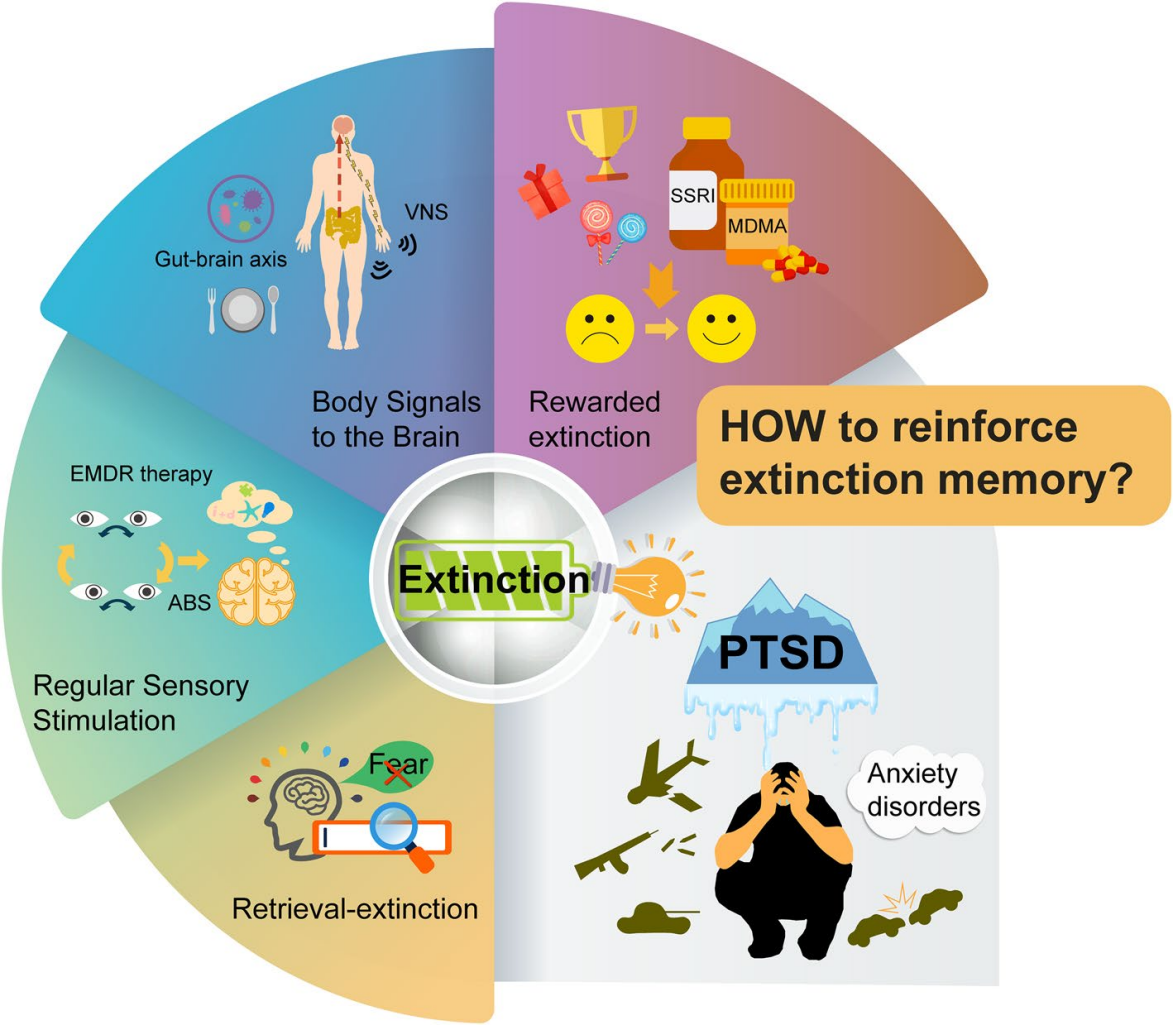
Schematic representation of behavioral strategies for reinforcing fear extinction memory. (1) Retrieval-extinction procedure to destroy the original fear memory trace. (2) Regular sensory stimulation for enhancing fear extinction. EMDR, eye movement desensitization and reprocessing; ABS, alternating bilateral sensory stimulation. (3) Body signals to the brain contribute to fear extinction. VNS, vagus nerve stimulation. (4) Rewarded extinction stabilizes the long-term extinction memory trace. MDMA, 3,4-methylenedioxymethamphetamine; PTSD, post-traumatic stress disorder; SSRI, serotonin selective reuptake inhibitor. Please see the text for more details

One can destroy the original fear memory trace to attenuate conditioned fear behaviors by targeting a process termed reconsolidation [110]. A previously consolidated memory becomes labile and prone to disruption or is modified after retrieval, requiring reconsolidation to restabilize the reactivated/destabilized memory [111, 112]. As a result, the reconsolidation process allows memories to be updated with new information available during retrieval [110, 112, 113]. In the auditory fear memory paradigm, Monfils *et al* developed a behavioral procedure in which an isolated retrieval trial is presented to induce lability of the original fear memory prior to extinction training, resulting in a permanent attenuation of the fear memory without return of learned defensive responses [114], suggesting that the original fear memory has been modified. This retrieval-extinction procedure (Fig. 4) in rodents to alter fear memory was quickly translated to humans, as extinction training during reconsolidation prevented the return of defensive responses for at least one year, consistent with permanent changes in fear memory [115]. Notably, neuroimaging studies of the retrieval-extinction paradigm showed less prefrontal cortical involvement compared to typical extinction [116], in accordance with the hypothesis that the amygdala-dependent fear memory was edited and, therefore, prefrontal inhibition was unnecessary. In the engram view, such fear attenuation caused by the retrieval-extinction procedure occurs through updating the original fear trace toward safety by increasing overlap between fear and extinction engram neurons, ultimately erasing fear memory [117]. This fear attenuation can also be observed in the remote fear memory scenario [118], but the attenuation of remote fear memory depends on a specific thalamo-amygdalar circuit [119, 120].

In order to expand the application of trauma cues in the clinical setting, with the aim of targeting and modifying maladaptive memories associated with PTSD, Ressler *et al* developed a novel procedure known as backward fear conditioning [121]. This procedure allowed for the indirect retrieval and manipulation of a contextual fear memory engram in rats, which is dependent on the hippocampus [121]. Through this approach, the researchers discovered that conditioned freezing in response to a backward CS was mediated by fear of the conditioning context [121]. This fear response activated specific ensembles within the hippocampus, which could be covertly captured and chemogenetically activated to elicit fear [121]. These findings support that in a clinical context, interventions targeting traumatic memories may benefit from indirect retrieval methods, such as imaginal exposure, to create an opportunity for modifying or even erasing neural representations associated with pathological fear.

### Regular Sensory Stimulation for Enhancing Fear Extinction

Translating from psychotherapeutic methods that use visual stimulation, eye movements, or attentional control of cognitive processes to produce long-lasting fear attenuation, researchers sought to uncover the role of regular sensory stimulation in enhancing fear extinction. For example, in a psychotherapeutic regimen termed eye movement desensitization and reprocessing (EMDR), patients are instructed to recall a traumatic memory while orienting to alternating bilateral sensory stimulation (ABS) [122]. To elucidate the neural basis of ABS, Baek *et al*. induced a lasting reduction in fear in mice by pairing visual ABS with CS during fear extinction and identified a neural pathway driven by the superior colliculus (SC)—which has been involved in visual-attentional processing [123, 124]—that mediates persistent attenuation of fear [125]. ABS produced the strongest fear-reducing effect, resulting in a sustained increase in SC and mediodorsal thalamus (MD) activity, with the SC-MD circuit being critical in preventing the return of fear. ABS stabilized inhibitory neurotransmission in the BLA through a feedforward inhibitory circuit from the MD, thereby inhibiting the activity of fear-encoding cells. Taken together, these findings suggest an interaction between sensory and fear memory circuits to mediate regular sensory stimulation for sustainable attenuation of traumatic memories (Fig. 4).

### Body Signals to the Brain Contribute to Fear Extinction

A novel approach to balancing fear and extinction of memories involves the integration of peripheral afferent information into the brain through body-brain interaction. While fear triggers strong bodily responses, such as changes in heart and breathing rates [126–129], these bodily feedback signals also play a crucial role in emotion regulation [130–133], including responsiveness to extinction training. The insular cortex (IC) serves as a core region involved in processing bodily signals and receives inputs from distinct thalamic and brainstem nuclei, which transmit visceral and cardiovascular signals from the periphery to the brain [134, 135]. A study by Alexandra *et al*. demonstrated that the IC integrates predictive sensory and interoceptive signals to provide graded and bidirectional teaching signals that control fear extinction, highlighting how bodily feedback signals are utilized to maintain fear within a functional balance [136]. Disrupting this balance between fear extinction and maintenance, similar to inhibiting the IC, can be achieved through vagus nerve stimulation (VNS), revealing the importance of body-brain communication [136]. VNS, as a neuromodulation therapy strategy, has also been shown to enhance motor learning through cholinergic signaling [137], indicating the presence of unidentified circuit modulation underlying its potential effects on fear extinction. Furthermore, a noninvasive optogenetic pacemaker has been developed to precisely control heart rhythm, resulting in optically induced tachycardia, which increases anxiety-like behavior specifically in risky contexts [138]. The posterior IC may serve as a potential mediator of bottom-up cardiac interoceptive processing, suggesting that both central (brain) and peripheral (body) processes together are required for the development of emotional or affective states [138].

Regarding the central cellular mechanisms responsible for body-brain communication, our research identified two distinct subpopulations of projection neurons located in close proximity within the IC. These subpopulations target the CeA and nucleus accumbens (NAc), respectively, and encode fear and extinction memories [22]. However, it remains to be determined whether and how the IC-CeA and IC-NAc projectors, along with other unidentified ensemble populations, differentially contribute to the balanced control of extinction and maintenance of fear memory through bodily feedback.

In addition, it has been observed that engaging in acute mild exercise prior to extinction training can improve recent and remote retention of fear extinction [139]. Furthermore, a study has indicated that alterations in microbiota composition have a significant impact on fear extinction learning [140]. This suggests that peripheral information, such as diet, infection, and lifestyle, play a role in shaping brain health and determining susceptibility to neuropsychiatric disorders (Fig. 4). Overall, various internal physiological states [141, 142], which are bottom-up manifestations of homeostatic processes and have widespread effects on the organism’s body, including metabolic factors (hunger, satiety), arousal-related factors, and immunological states, can induce a specific brain state that inevitably influences future physiology and/or behavior, such as fear extinction. Further extensive research is needed to explore the mechanisms of fear extinction regulated by signals from the body to the brain.

### Rewarded Extinction Stabilizes the Long-term Extinction Memory Trace

As discussed above, fear extinction involves reward circuitry [65, 68, 91, 143], so incorporating reward associations with a fear extinction memory is likely to be an effective strategy for persistently attenuating fear responses. Indeed, rewarded extinction, exemplified as counterconditioning in rats enhanced recruitment of an amygdala-striatal pathway and led to reduced fear relapse in the spontaneous recovery test [144], but exhibited greater levels of fear renewal [145]. Consistent with these observations in rodents, replacing shock with reward, rather than merely omitting it, generated a more stable and enduring memory trace of extinguished fear in humans [146]. As a promising pharmacological approach to enhance reward processing [147], 3,4-methylenedioxymethamphetamine (MDMA)-assisted psychotherapy has long-lasting therapeutic effects on traumatic memories for PTSD [148]. MDMA has also been shown to enhance fear extinction [149] and modulate fear memory reconsolidation [150]. Similar to MDMA, which acts as a serotonin transporter inhibitor to induce serotonin release [151, 152], fluoxetine, a serotonin selective reuptake inhibitor (SSRI) antidepressant, along with extinction training, also promotes fear extinction to a state of fear erasure in mice, likely by converting the fear memory circuitry to a more immature state *via* local BDNF [153]. These findings suggest that incorporating reward either behaviorally or pharmacologically enhances fear extinction and that reward may be a valuable adjunct to exposure-based therapies for PTSD and other anxiety disorders characterized by altered fear learning (Fig. 4).

## Conclusion and Perspectives

Fear extinction is not only a typical form of inhibitory learning but also a translational model for psychological exposure therapy for many emotional disorders, such as PTSD and anxiety disorders. Inherently, extinction training modifies the original fear memory trace and creates a new memory trace to control conditioned fear behaviors. Here, we have reviewed the memory trace at different levels for fear extinction that underlies the dynamic competition between fear and extinction memories in adaptive control of conditioned fear responses. The configuration of multilevel neural circuit plasticity conferring memory trace for fear extinction, which is centered around the tripartite engram construct consisting of the amygdala, mPFC, and vHPC, deserves further investigation, along with its projection to other brain regions such as thalamic regions, IC, NAc, and other emerging brain regions. Although inevitably fragile, the new memory trace established during fear extinction can be strengthened by appropriate stimulation and manipulation, facilitating the development of therapies for PTSD and anxiety disorders.

A number of questions concerning this fragile yet reinforceable memory trace for fear extinction warrant further investigation. *First*, the dynamic nature of the memory trace in the longitudinal transformation of fear extinction memory remains to be further elucidated. In contrast to the original fear memory, fear extinction memory is more readily forgettable than the original fear memory. Based on the modern notion [92] that forgetting is a form of neuroplasticity that alters the accessibility of engram cells in a manner that is sensitive to mismatches between expectations and the environment, how can the failure of fear extinction memory retrieval be explained on a case-by-case basis? Regarding the spontaneous recovery of the extinguished fear, there is an urgent need to comprehensively study the dynamic evolution of fear and extinction memories, as a single fear memory undergoes extensive circuit reorganization for memory retrieval. As Caroni suggested for the next generation of neuroscience for the neuronal assemblies and memory, there are currently two approaches by which to study learning-related neuronal assemblies [154]. One type addresses the dynamics of activity in large sets of neurons with, for example, repeated calcium imaging experiments in behaving animals. The other type addresses the functional roles of “memory neuron” assemblies using genetic tagging and/or manipulation experiments of learning-related “memory neurons” based on the expression of activity-regulated genes (Fos, Arc). What remains to be done in the future is to develop conceptual frameworks to relate the resulting findings to each other in the context of fear extinction.

Second, like other forms of memory, cellular compositions of engram complexes for fear extinction need to be continually updated. On one hand, the functional heterogeneity within the individual fear extinction memory engram needs to be clarified. It has been suggested that contextual fear memory engrams in the mouse dentate gyrus contain functionally distinct neuronal ensembles, genetically defined by the Fos- or Npas4-dependent transcriptional pathways, that promote memory generalization and discrimination, respectively [155]. It is known that omission of punishment, switching from negative to positive emotional valence, and contextual processing are necessary components of fear extinction, but the neuronal ensembles responsible for each component remain to be identified in the future. On the other hand, in addition to engram neurons, various types of glial cells are also likely to contribute to fear extinction engram complexes. In another phase of fear memory, microglia have been shown to mediate forgetting *via* complement-dependent synaptic elimination [156]. Pathologically, modulation of oligodendrocyte myelination can affect cognition in the mouse model of Alzheimer’s disease [157]. The tetrapartite synapse model, which includes neuronal pre- and post-synaptic terminals, the extracellular structural scaffold together with the cellular glue (i.e., glia), neurovascular unit (NVU), and immune systems, is thought to play a role in long-term plasticity and circuit maintenance [158]. The cellular and molecular aspects of neuron-glia crosstalk in the scenario of fear extinction need to be identified, in particular the interactions between different types of glial cells and engram neurons to regulate synaptic communication [159].

Third, how to understand fear extinction in terms of the organization of different memories [160], or the mechanisms underlying the dynamic nature of fear extinction influenced by other cognitive experiences. It has been proposed that memories sharing certain attributes are known to interact so that retrieval of one increases the likelihood of retrieving the other, raising the possibility that related memories are organized into associative mnemonic structures of interconnected representations [161–163]. Fear extinction is a potential model for investigating the neural mechanisms that organize and link related memories, including the original fear memory and related contextual memories. A deep understanding of the molecular, cellular, and systems mechanisms that support the organization of memories along dimensions of time, space, and perceptual/conceptual similarities [160] will undoubtedly help understand and manipulate the complex and intricate factors for the expression of fear extinction. In addition, by linking mnemonic structures and the integration of previous and current memories, the experience of successful fear extinction can be extended to more general emotional resilience, the ability to overcome the negative experience, which may help to ameliorate emotional disorders.

Finally, from a translational perspective, the effective strategies for reinforcing memory traces of fear extinction are far from being satisfactory for clinical needs. By targeting specific circuits for fear extinction, nerve stimulation approaches including VNS, repetitive transcranial magnetic stimulation (rTMS), and deep brain stimulation (DBS) exhibit promising but not reliable therapeutic effects in both animal models and clinical patients like PTSD [164, 165]. The specific neural mechanisms behind these neural stimulation approaches merit further investigation to optimize more specific treatment regimens. In addition, the identification of molecularly defined extinction-specific cellular ensembles and molecular substrates that orchestrate valence assignment [166] should inform new strategies for the development of pharmacological therapies that target extinction-related symptomatology associated with numerous neuropsychiatric disorders, including PTSD and anxiety disorders.
